# Antiosteoporosis Studies of 20 Medicine Food Homology Plants Containing Quercetin, Rutin, and Kaempferol: TCM Characteristics, *In Vivo* and *In Vitro* Activities, Potential Mechanisms, and Food Functions

**DOI:** 10.1155/2022/5902293

**Published:** 2022-03-31

**Authors:** Dayue Shen, Yating Feng, Xilan Zhang, Le Gong, Jing Liu, Yuanping Li, Hui Liao

**Affiliations:** ^1^School of Pharmacy, Shanxi Medical University, Taiyuan 030001, China; ^2^Department of Pharmacy, Fifth Hospital of Shanxi Medical University (Shanxi Provincial People's Hospital), Taiyuan 030012, China

## Abstract

Dietary nutraceutical compounds have been evidenced as backbone for bone health in recent years. It is reported that medicine food homology (MFH) plants have multiple nutraceutical compounds. Based on our literature research, 20 MFH plants caught our attention because they contain three popular antiosteoporosis compounds simultaneously: quercetin, rutin, and kaempferol. According to traditional Chinese medicine (TCM), their characteristics including natures, flavors, attributive to meridian tropism, and efficacies were listed. The relationships between TCM efficacies, such as “heat clearing,” “tonic,” and “the interior warming,” and antiosteoporosis pharmacological actions such as antioxidant and immune regulation were discussed. The *in vivo* antiosteoporosis effects of the 20 MFH plants were summarized. The *in vitro* antiosteoporosis activities and related mechanisms of the 20 plants and quercetin, rutin, kaempferol were detailed. The TGF-*β*-Smad signaling, fibroblast growth factor, and Wnt/*β*-catenin signaling on bone formation and the RANKL signaling, NF-*κ*B signaling, and macrophage-colony-stimulating factor on bone resorption were identified. From food point, these 20 MFH plants could be classified as condiment, vegetable, fruit, tea and related products, beverage, etc. Based on the above discussion, these 20 MFH plants could be used as daily food supplements for the prevention and treatment against osteoporosis.

## 1. Introduction

Osteoporosis is a systemic metabolic bone disease characterized by bone mass decrease and microstructural degradation, which may increase the risk of bone fracture and lead to high mortality [1]. China National Health Commission conducted an osteoporosis epidemiological survey in 2018. The results showed that osteoporosis has become an important health problem for middle-aged and elderly people in China: the incidence of osteoporosis was 19% in people over 50 years old and even reached 32% in over 65 years old [2]. It is confirmed that factors such as age, sex, weight, and diabetes are significant predictors of osteoporosis in the Chinese people [3]. Estrogen deficiency and aging are the main causes for disturbances in bone remodeling activity and bone loss [4]. The current drugs for the treatment of osteoporosis include bisphosphonates, estrogen, receptor activator of nuclear factor kappa B ligand (RANKL) inhibitors, etc. These drugs play important roles clinically, but their serious side effects limit their clinical use [5, 6].

Scientific reports suggest that natural Chinese medicine therapies appear to have both the anabolic and anticatabolic effects for the treatment of osteoporosis by promoting bone formation and attenuating imbalanced bone resorption, leading to improved bone mineral density and reducing bone microstructural degradation. A wide range of natural compounds were identified to bear this potential. The identified natural compounds are summarized such as kaempferol, icariin, and berberine [7]. The classic and bone-specific drugs in natural Chinese medicines for the treatment of osteoporosis were reviewed comprehensively on the treatment of osteoporosis that had been deeply and definitely studied [8]. These studies provide a critical overview of alternative medicine for the treatment and prevention of osteoporosis.

Dietary nutraceuticals as backbone for bone health in Chinese medicine therapies have been evidenced in recent years. The nutraceutical compounds such as ginsenosides and quercetin identified from medicinal plants can reverse/slow down osteoporosis. Most of these compounds are inexpensive and have no side effect [9]. Some nutraceutical compounds are from medicine food homology (MFH) plants, such as ginsenosides from ginseng [10].

“The list of MFH species” was updated by the National Health Commission in 2014, and a total of 94 MFH plants were included in this list [11]. Compared with modern dietary nutrition, MFH plants have unique beneficial concepts, such as the holism, and diet suggestions based on different syndromes. On the other hand, the MFH research has some limitations such as lack of evidence-based data and difficult to evaluate the active ingredients [12]. From the point of pharmacological research, some nutraceutical compounds, mainly including quercetin, resveratrol, curcumin, rutin, and kaempferol [9], showed more and more antiosteoporosis evidences recently.

**Figure 1:**
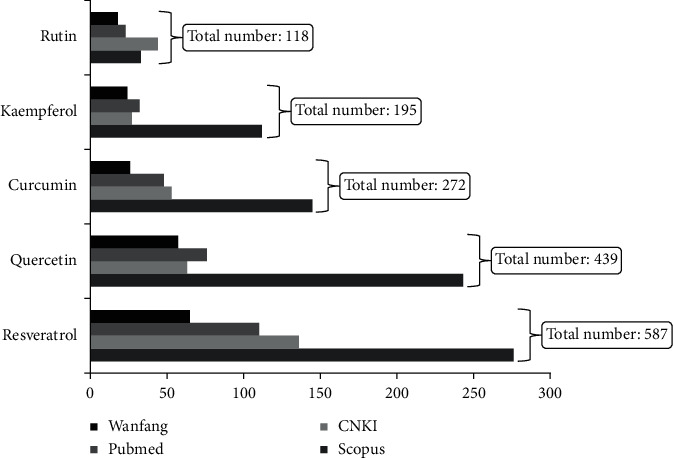
Antiosteoporosis literature research of five popular nutraceutical compounds [9]. Abbreviations: CNKI, China National Knowledge Infrastructure; Wanfang, Wanfang Database.

**Figure 2:**
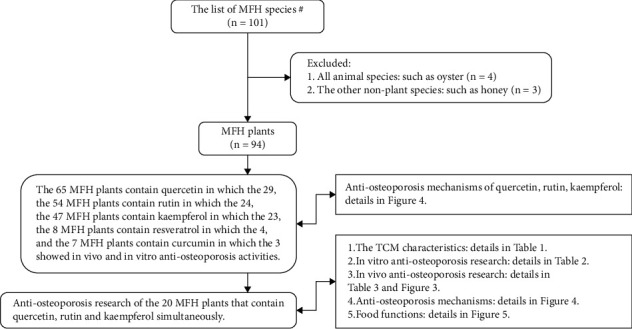
Antiosteoporosis research of the 20 MFH plants that contain quercetin, rutin, and kaempferol. *Notes*. ^#^The list of MFH species is according to “The management about medicine food homology catalog” issued by the National Health Commission of the People's Republic of China in 2014 [11]. Abbreviations: MFH, medicine food homology; TCM, traditional Chinese medicine.

**Figure 3:**
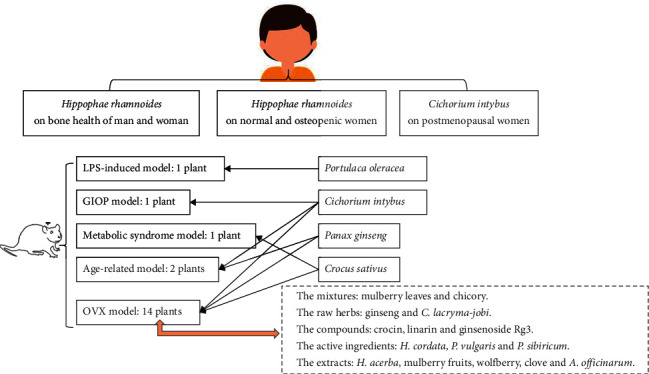
*In vivo* clinical and animal research of medicine food homology plants. Abbreviations: LPS, lipopolysaccharide; GIOP, glucocorticoid-induced osteoporosis; OVX, ovariectomy.

**Figure 4:**
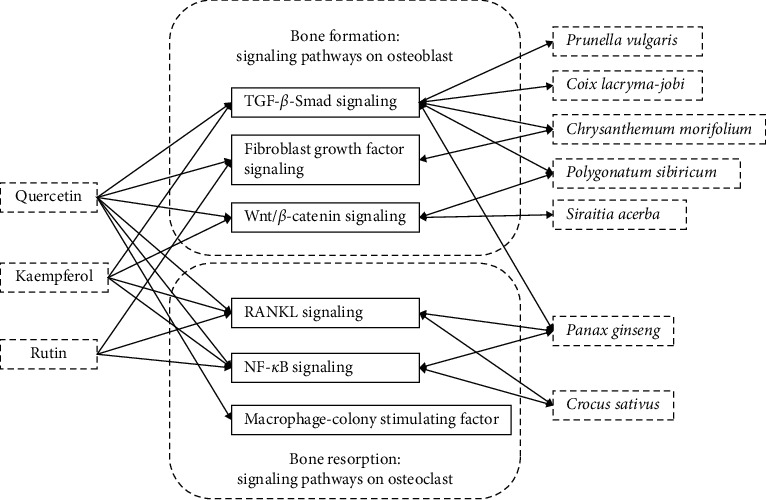
Mechanism research of quercetin, kaempferol, rutin, and medicine food homology plants on bone formation and bone resorption. Abbreviations: TGF-*β*, transforming growth factor-*β*; Smad, suppressor of mothers against decapentaplegic; Wnt, wingless; RANKL, receptor activator for nuclear factor kappa B ligand; NF-*κ*B, nuclear factor kappa B.

**Figure 5:**
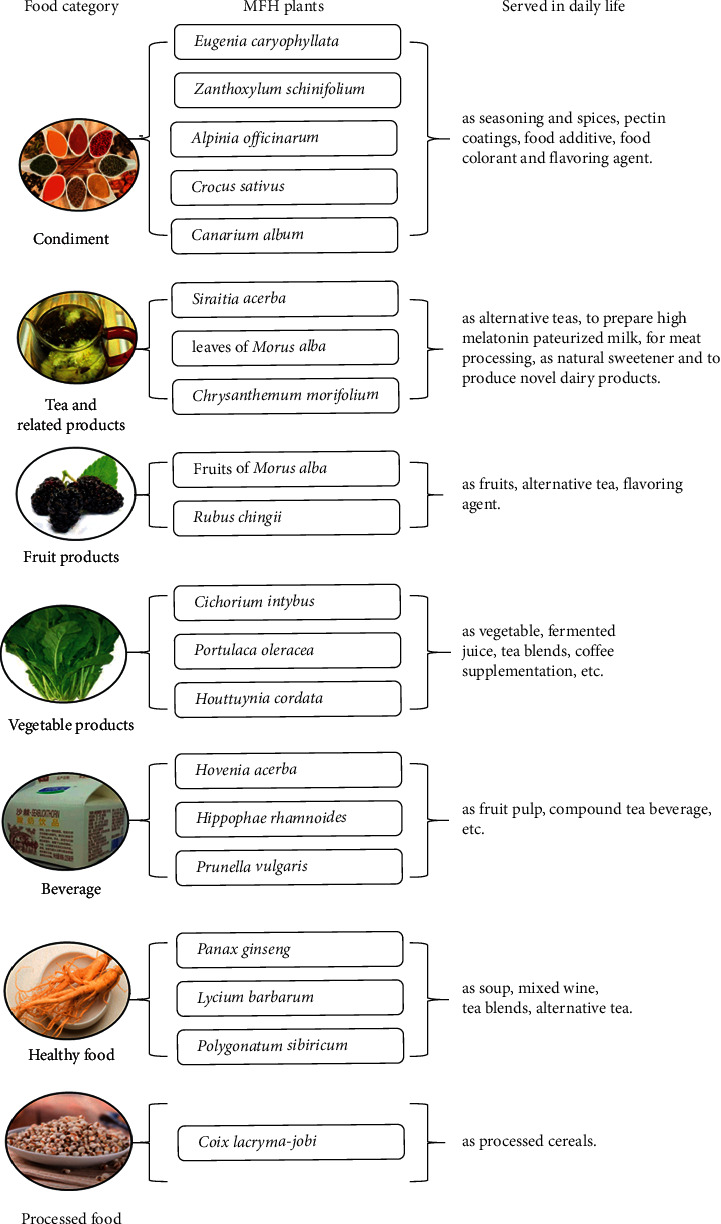
Food functions of the 20 MFH plants. Abbreviation: MFH, medicine food homology.

Based on PubMed, Scopus, Wanfang, and CNKI database, the literature of the five popular nutraceutical compounds [9] on antiosteoporosis effects was searched, and the results showed that most of the relevant studies are on resveratrol, quercetin, and curcumin (Figure 1). Further literature studies showed an interesting result: quercetin, rutin, and kaempferol were present in more MFH plants than curcumin and resveratrol (Figure 2, the 38 references did not show). Based on above information, the 20 MFH plants that contain quercetin, rutin, and kaempferol simultaneously were discussed in this manuscript. Their characteristics according to the theory of traditional Chinese medicine (TCM) are summarized in Table 1, the *in vitro* antiosteoporosis research is detailed in Table 2, and the *in vivo* antiosteoporosis research is detailed in Table 3 and Figure 3. Their antiosteoporosis mechanisms related to quercetin, rutin, and kaempferol are identified in Figure 4. This review finally explored the possibilities of the 20 MFH plants as antiosteoporosis food supplementation, as shown in Figure 5.

## 2. TCM Characteristics

### 2.1. Four Natures and Five Flavors

The properties and actions of Chinese herbs are mainly summarized as the four natures and five flavors, meridian tropism, and toxicity. Four natures and five flavors are also known as the properties and tastes of Chinese herbs. Cold-cool and hot-warm are two completely different categories of natures [55]. As shown in Table 1, there are ten herbs in the cold-cool category and six in the hot-warm category. In addition, there are also some herbs known as neutral ones whose cold or hot nature is not so remarkable and whose action is relatively mild. Among the 20 MFH plants, there are four herbs with neutral properties.

The five flavors of Chinese herbs refer to the five different tastes: pungent, sweet, sour, bitter, and salty [56]. Actually, the five flavors are not only the true reflection of the taste of drugs but also the high generalization of the effects of herb medicines. Since the theory of five tastes was applied to summarize therapeutic effects, the “taste” of five tastes has gone beyond the scope of the real taste of herb medicines. From Table 1, we could see that there are 9 MFH plants with sweet taste (alone or dominated), 5 MFH plants with pungent taste, 2 MFH plants with sour, and 4 MFH plants with bitter taste.

### 2.2. Attributive to Meridian Tropism

Meridian tropism refers to that herbs often produce their therapeutic effects on some portion of a human body in preference. If certain herb can work on several meridians, which means the herb can be used widely to treat the disorders of these meridians. From Table 1, we could see that only 2 MFH plants point a single meridian, and the other 18 MFH plants have more than two meridians. The meridian tropisms include spleen and liver (10 MFH plants, separately), lung (9 MFH plants), stomach (7 MFH plants), and kidney (6 MFH plants).

In TCM, kidney deficiency is the main pathogenesis of osteoporosis based on the theory of “kidney governing bones” [57]. On the other hand, modern clinical and preclinical research showed that liver-spleen-kidney insufficiency may result in the development of diabetic osteoporosis [58]. In this case, it is necessary to develop an objective and comprehensive method to evaluate and understand the antiosteoporosis effects of the 17 MFH plants attributed to the liver, spleen, and kidney.

### 2.3. Traditional Efficacies and Modern Antiosteoporosis Actions

According to some research, there was a certain relationship between TCM efficacies and modern pharmacological actions [59]. In order to find the relationships between traditional efficacies of the 20 MHF plants such as “heat clearing” and “diaphoretics with cool property” and modern antiosteoporosis pharmacological action, we will provide some information and references as follows.

“Clearing away heat” in TCM displays a variety of biological activities and mainly antioxidant and anti-inflammatory actions [59, 60]. Attenuating effect of *P. oleracea* extract on chronic constriction injury-induced neuropathic pain in rats was related to its antioxidative and anti-inflammatory effects [61]. *H. cordata* crude extract administration inhibited fever in rats, reduced the number of leukocytes, and restored serum complement levels [62]. A recent study demonstrated a broad range of biological activities of *P. vulgaris*, including immune modulatory, antiviral, anti-inflammatory, antioxidant, and antidiabetic [63]. Chemical compositions and antioxidant activities of *C. album* were identified, and dietary intakes of health-promoting components were also estimated [64]. The antioxidant activities, antiglycation effects, and inhibition activities on *α*-glucosidase and *α*-amylase of seven extracts from *H. acerba* were confirmed *in vitro* [65]. The health-promoting activities attributed to *C. intybus* include anti-inflammatory, antimutagenic, antifungal, and antioxidative qualities [66].

Chinese herbs “with cool property” also show important pharmacological effects on antioxidant activities [67]. Mulberry leaves extract ameliorated alcohol-induced liver damages through reduction of acetaldehyde toxicity and inhibition of apoptosis induced by oxidative stress signals [68]. Antioxidant effects of *C. morifolium* could be potent phytochemical agents to reduce low-density lipoprotein (LDL) oxidation and prevent the progression of atherosclerosis [69].

Traditional “tonic” efficiency is normally related with immune regulation effects [70]. Ginseng has been used worldwide for its miracle “tonic” effects, especially for its immunomodulatory activities [71]. The fruits of *M. alba*have been traditionally used as a “tonic” to enhance immune responses [72]. *P. sibiricum* participated in the protection against immunosuppression in cyclophosphamide-treated mice, highlighting its potential as an immunostimulant [73]. Several clinical studies in healthy subjects showed that consumption of wolfberry juice improves general wellbeing and immune functions [74]. It is reported that antiosteoporotic activity is related to the regulation of immune functions and antioxidant activity [75]. In this case, traditional “tonic”, “clearing away heat” efficiency, and “with cool property” could explain the antiosteoporotic activity from the point of antioxidant and immune-regulatory activities.

It is interesting that “the interior warming” efficiency could be understood from the point of penetration enhancement activity of essential oils from *E. caryophyllata*, *Z. schinifolium*, and *A. officinarum* [76]. Flurbiprofen is one of the most potent nonsteroidal anti-inflammatory drugs with very low bioavailability of approximately 12% after transdermal administration, compared with that after oral administration. The essential oils from *A. officinarum* could be used as an oil phase and a penetration enhancer that help transdermal delivery of flurbiprofen [77]. Further study in ovariectomy (OVX) rats showed that osteogenic efficacy can be enhanced by kaempferol through an engineered layer-by-layer matrix [78]. That might explain the antiosteoporosis effect of “the interior warming” efficiency based on penetration enhancement activity of essential oil and kaempferol.

From a modern perspective of the cardiovascular system, it seems easy to understand the ancient efficiency of “activating blood circulation and removing blood stasis.” In TCM, the antiosteoporotic effect of some traditional herbs is ascribed to their action on liver and blood stasis as main therapeutic targets defining osteoporosis [79], but the research of MFH plants with “removing blood stasis” efficiency on antiosteoporosis effect should be conducted further.

For more than 300 years, *S. grosvenorii* has been used as a natural sweetener and as a traditional medicine for the treatment of pharyngitis and pharyngeal pain, as well as an antitussive remedy in China [80]. Its ancient antitussive efficiency is similar to modern terms but difficult to understand from the antiosteoporosis point. The mechanisms of adlay were confirmed in regulating the water transport on the spleen deficiency and wet dampness rat model [81]. It is also hard to understand its antiosteoporosis effects from the point of “dampness removing.”

Based on the above discussion, some traditional efficacies of MFH plants might be related with modern antioxidant and immune-regulatory activities, and latter are associated with antiosteoporosis effects.

## 3. *In Vivo* Clinical Studies

Postmenopausal women tend to be susceptible to primary osteoporosis due to its association with estrogen deficiency. Aside from physical activity, nutrition and diet in adequate proportions are suggested to be an important tool for ameliorating osteoporosis and bone health issues in postmenopausal women [82]. There were three clinical studies that correlated with postmenopausal women [83–85].

The first clinical study was a test of a mixture of chicory oligofructose and long-chain inulin on 15 postmenopausal women ((72.2 ± 6.4) years). The women were treated with the mixture for 6 weeks using a double-blind, placebo-controlled, crossover design [83].

The second one was a study about sea buckthorn oil fatty acid, which was tested on normal and osteopenic women. A total of 1865 female subjects (20–79 years old) were enrolled in this study. The results showed that the intake of the oil fatty acids seemed to be positively associated with bone mineral density (BMD), which is obtained by dividing the bone width by the bone mineral content (BMC) at both the hips and the lumbar spine in normal and osteopenic women [84].

The last one was about omega-3 polyunsaturated fatty acids from sea buckthorn, which was studied on bone health of 20 males and 3 females for 6 weeks in a randomized, 3-period crossover design. The results indicated that sea buckthorn may have a protective effect on bone metabolism via a decrease in bone resorption in the presence of consistent levels of bone formation [85].

In summary, clinical antiosteoporosis studies of the 20 MFH plants are still limited. The popular compounds such as quercetin and kaempferol have not reported clinical research till now [9]. Well-designed clinical studies will help discover the antiosteoporosis evidences of these MFH plants and also compounds.

## 4. *In Vivo* Animal Studies

### 4.1. OVX Models

Animal models of osteoporosis are appropriate tools for establishing new prevention strategies and more effective treatment modalities, of which the OVX rat model is the most commonly used [86]. From Figure 3, we could see that the 14 MFH plants were tested on OVX animal models and they all had positive effects on bone health. These 14 MFH plants were tested on mixtures [41, 54], single herbs [18, 30, 37, 45, 47, 50, 51], and active ingredients [19, 32, 39, 40, 48, 52] separately.

The combined extract of mulberry leaves and *Polygonum odoratum* were also tested [54]. Chicory inulins and a mixture of inulins-isoflavones were compared in OVX rats. The results showed that there were no apparent histological changes in rats treated with inulins and the mixture [41].

The tested two raw herbs were ginseng [45] and adlay [37]. The tested extracts were from *H. acerba* [18], mulberry fruits [47], wolfberry [50], clove [51], and *A. officinarum* [30].

The tested active ingredients included the essential oil of *H. cordata* [39], the flavonoids from *P. vulgaris* [40], and the polysaccharide from *P. sibiricum* [48]. The tested nutraceutical compounds included crocin from saffron [52], linarin from chrysanthemum [32], ginsenoside Rg3 from ginseng [19], etc.

Based on OVX models, the effects of *P. sibiricum* were reported on an OVX-induced fracture model that was established by broken femoral shaft in OVX rats. The related research showed that *P. sibiricum* improved the biomechanical properties and BMD of fracture rats by regulating bone repair and bone metabolic factors [49]. Most studies have reported that polysaccharides, flavonoids, and saponins are the main bioactive compounds in *P. sibiricum*, which play important roles on antioxidation [87]. That suggested that the antifracture effect of *P. sibiricum* might be related with its antioxidation activities.

*In vitro* cultured rat femoral metaphyseal tissues isolated from an OVX model were used for the research of adlay extract. The results showed that adlay seeds could reverse the decreased calcium levels induced by parathyroid hormone in cultured metaphyseal tissues [38].

### 4.2. Age-Related Models

Bone health is important throughout the whole human life: infancy, adolescence, adulthood, and old age. Age-related model studies in this review included a nutraceutical compound from chicory on growing rats [42] and young rats [43] and extracts and compounds from ginseng on aged animals [19, 46, 88].

Inulin, a nutraceutical compound from chicory, showed its effects on whole-body BMC, whole-body bone area, and whole-body BMD in growing male rats (4-5 weeks old). The results also showed that chicory inulin not only increases calcium absorption but also increases mineral parameters in whole-body bones [42].

Furthermore, a purified native inulin, a reformulated inulin based on a combination of short- and long-chain fructans, and a dehydrated chicory were tested in 3-month-old young male rats, and the experiment was continued for 3 months. The results showed that bone parameters had a significant positive improvement by the chicory diet, whereas the purified inulin diets were less effective. The particular effects of the chicory crude fraction on digestive fermentation and bone parameters suggest possible synergisms between inulin-type fructans and other nutrients [43].

It is well known that aging leads to impaired bone regulation, resulting in an imbalance between bone homeostasis and pathological bone mass [89]. Using natural 112-week-old male rats, some research indicated that the extracts from ginseng might be a potential alternative medicine for the prevention and treatment of natural aging-induced osteoporosis in humans [46]. Ginsenosides are pharmacologically active compounds that are often extracted from *P. ginseng* for their medicinal properties. Many ginsenosides can promote bone formation and inhibit bone resorption, such as Rb1, Rb2 [88], and Rg3 [19]. We are interested that these ginsenosides have effects on aging-induced osteoporosis in the following research.

As we discussed before, estrogen deficiency and aging are the main causes for disturbances in bone remodeling activity and bone loss [4]. The current drugs for the osteoporosis include estrogen treatment [5, 6]. Estrogen is involved in the regulation activity of osteoblasts and osteoclasts, directly through estrogen receptor (ER)-*α* and ER-*β*. In the last decades, *in vitro* and *in vivo* results have revealed that *P. ginseng* and its active compounds are equipped with hormone analogous effects. There is evidence that ginsenosides Rg1 exert estrogen-like effect via the activation of ER-*α* [90].

### 4.3. Glucocorticoid-Induced Models

Glucocorticoid-induced osteoporosis (GIOP) that is mainly featured as low bone density and increased risk of fracture is prone to occur with the administration of excessive glucocorticoids [91]. Chicory showed bone protection against GIOP in rats, and the protective effects were related to its flavonoids and inulin [44]. Estrogen is involved in the regulation activity of bone, indirectly through parathyroid hormone (PTH). The chicory extracts can decrease PTH in GIOP rats [44].

### 4.4. Metabolic Syndrome Models

Metabolic syndrome is a serious health problem. Complications of metabolic syndrome include osteoporosis. Antiosteoporotic activities of crocin from saffron were evaluated in a rat model of metabolic syndrome-induced osteoporosis. Crocin enhanced both the longitudinal and perpendicular bone strength (bone strength means the ratio of the maximum load value to the BMC per millimeter of specimen length) of femurs. That enhanced effects of crocin mitigated oxidative stress in femur distal epiphysis tissues [53].

### 4.5. LPS-Induced Bone Loss Model

Injection of lipopolysaccharide (LPS) rapidly induced trabecular bone loss through stimulation of osteoclast differentiation. The protective effect of purslane on LPS-induced bone loss was confirmed. Purslane exhibited its antiosteoclastogenic activity based on its anti-inflammatory and antioxidative properties [13].

## 5. *In Vitro* Studies

The homeostasis of bones is jointly maintained by osteoblasts, osteoclasts, bone marrow mesenchymal stem cells (BMMSCs), and other cells [92]. Osteoporosis is produced by an imbalance between osteoblastogenesis and osteoclastogenesis processes during bone metabolism. Inflammation and high reactive oxygen enhance osteoclastogenesis while reducing osteoblastogenesis by inducing osteoblast apoptosis and suppressing osteoblastic proliferation and BMMSC differentiation [93].

### 5.1. On Osteoblastogenesis Processes

A total of 12 MFH plants were reported to have a positive regulatory effect on osteoblast differentiation. The extracts from *H. acerba* [18], *A. officinarum* [30], and mogroside V from *S. grosvenorii* [33]. The above three samples were tested on calvarial osteoblasts from the rat/mice calvaria at postnatal day. The polysaccharides from *L. barbarum* [28], and three compounds including crocin [34], linarin via bone morphogenetic proteins 2(BMP-2)/runt-related transcription factor 2（Runx2） pathway [42], and ginsenoside Rb2 via a reduction of oxidative damage [20] were all tested on the osteoblast MC3T3‐E1 cell line. The results showed that osteoblast differentiation could be induced by the above extracts and compounds [18, 20, 28, 30, 33, 34, 42].

Comparison of seven compounds from *R. chingii* was carried out on primary rat osteoblasts [29]. Purslane was tested on primary human osteoblasts [14], and the adlay extract was tested on primary rat osteoblasts isolated from the calvaria of fetal rats (18 days old) via an extracellular signal regulated kinase (ERK) pathway [37]. The promoting effects on osteoblast differentiation could be seen in the related results [14, 29, 37].

The effects of two MFH plants and their active ingredients on BMMSCs were reported. *P. vulgaris* protected glucocorticoids-induced osteogenesis inhibition in BMMSCs through activating the suppressor of mothers against decapentaplegic (Smad) pathway [16]. The polysaccharide from *P. sibiricum* was tested on primary mice BMMSCs [25, 26]. Crocin and crocetin, two major compounds from saffron, were compared and their effects on osteogenic differentiation of BMMSCs isolated from rats were tested [35]. Ginsenoside Rb1 from ginseng was reported to alleviate aluminum chloride-induced rat osteoblast dysfunction [21].

### 5.2. On Osteoclastogenesis Processes

Osteoclasts are functional cells and play a major role in pathological bone resorption. They are derived from hematopoietic precursors and undergo a series of differentiation and fusion steps in response to various humoral factors. RANKL-induced osteoclast formation is considered as an important canonical pathway [94].

According to current studies, two MFH plants were only tested on RANKL-induced osteoclastogenesis processes *in vitro*: both *C. album* and *Z. schinifolium* were tested on the RANKL-induced RAW 264.7 cell line [17]. The two MFH plant extracts from purslane [15] and *A. officinarum* [31] and the polysaccharide from *P. sibiricum* [25, 27] were also tested on RANKL-induced osteoclastogenesis processes on primary bone marrow-derived macrophages (BMMs).

The active compounds research included the following: crocin by regulating c-Jun N-terminal kinase (JNK) and nuclear factor kappa B (NF-*κ*B) signaling pathways [36]; ginsenoside Rg3 via RANKL, JNK, and p38 mitogen-activated protein kinase (MAPK) pathways [22], ginsenoside Rb1 via NF-*κ*B and MAPKs pathways [23]; and ginsenoside Rb2 via NF-*κ*B and STAT3 [24] were all tested and their effects on RANKL-induced RAW264.7 cells were defined.

## 6. Mechanism Studies

Signaling pathways are key players in the commitment and differentiation of osteoblasts and osteoclasts. A number of studies have identified key signals in the process of osteogenesis. From Figure 4, it seems that three compounds have more signaling research than MFH plants. Among the three compounds, quercetin is the most used one in pathway studies: transforming growth factor-*β* (TGF-*β*)-Smad signaling, fibroblast growth factor (FGF) signaling, wingless (Wnt)/*β*-catenin signaling in bone formation and RANKL signaling, NF-*κ*B signaling, and macrophage colony-stimulating factor (M-CSF) in bone resorption.

### 6.1. TGF-*β*-Smad Signaling

The TGF-*β* superfamily members bind and signal through dual type I and II transmembrane receptors, containing serine/threonine kinase domains. Smad proteins play key roles in transmitting signals from receptor to nucleus. One of the members of TGF-*β* superfamily, BMPs is critical in osteogenesis. The activation of MAPKs including ERK, JNK, and p38 is regulated by BMP-2 in osteoblastic cells [9].

The interaction of BMP-2 and TGF-*β* with their respective receptors results in the upregulation of osteogenic genes and osteoclastogenic genes via activation of Smad proteins and/or inhibition of MAPKs. Quercetin may have positive or negative effects on the levels of BMP-2, TGF-*β*, and Smad protein in bone cells [95]. The research found that kaempferol increased cell growth, secretion of osteoblast growth factor, and the level of BMP receptor II in opossum kidney cells. Findings from this study implied that kaempferol stimulated kidney repair, which indirectly stimulates bone formation [5].

The 5 MFH plants showed their effects on TGF-*β*-Smad signaling: the polysaccharide from Siberian Solomon's seal rhizome on primary mice BMMSCs via ERK [25], the extracts from common selfheal spike on glucocorticoids-induced BMMSCs by activating the Smad pathway [16], the adlay extract on the primary rat osteoblasts via the ERK pathway [37], compound linarin from chrysanthemum flowers on the osteoblast MC3T3‐E1 cell line via BMP-2 [32], and the compound ginsenoside Rb1 from ginseng alleviated aluminum chloride-induced rat osteoblast dysfunction via the increased TGF-*β* and BMP-2 expression [21].

### 6.2. FGF Signaling

The FGFs are the family members of secreted polypeptides. FGFs bind to FGF tyrosine kinase receptors (FGFRs) and then take part in a number of biological events critical in endochondral and intramembranous ossification. It has been demonstrated that FGF activates runt-related transcription factor 2 (Runx2) by MAPKs pathways and thus helps bone formation [9].

The treatment of MC3T3-E1 cells with rutin can significantly increase the expression of Runx2 gene, and significant differences were found among groups in which different concentrations were used [96]. The majority of studies have reported osteoblastogenesis-activating effects of quercetin via increased Runx2 levels, with a few exceptions. These discrepancies may be related to the cell types and doses of quercetin used [95]. Studies have shown that chrysanthemum flowers activate the transcription factor Runx2 through MAPKs pathways, therefore promoting bone formation [32].

### 6.3. Wnt/*β*-Catenin Signaling

The canonical Wnt/*β*-catenin signaling pathway has a central regulatory role in bone metabolism. The activation of Wnt signaling leads to the expression of the Wnt-targeted gene Runx2, which is essential for osteoblast differentiation [95]. In unstimulated conditions, *β*-catenin is sequestered into a destruction complex consisting of axis inhibition protein 2 (Axin-2), casein kinase 1 alpha (CK1α), adenomatosis polyposis coli (APC), and glycogen synthase kinase-3 beta (GSK3*β*). Higher levels of *β*-catenin signaling upregulate the expression of genes implicated in the differentiation of osteoblasts [9].

Quercetin rescued LPS-induced impairment of osteogenesis in murine osteoblastic MC3T3-E1 cells by enhancing the protein levels of Wnt3 and *β*-catenin and decreasing the protein level of GSK3*β* [95]. The stimulation of SaOS-2 cells by kaempferol resulted in an increased activity of Wnt signaling responsive reporter construct, Axin-2, and, subsequently, stabilization of Wnt signaling-mediated transcription factor *β*-catenin, probably leading to the activation of Wnt-targeted genes for osteogenesis [97].

The polysaccharide from Siberian Solomon's seal rhizome was tested on primary mice BMMSCs. The results showed that the polysaccharide promoted the osteogenic differentiation of BMMSCs. This effect was due to the increased nuclear accumulation of *β*-catenin, resulting in a higher expression of osteoblast-related genes [25]. One of the ingredients in raisin tree seeds was reported to activate the Wnt/*β*-catenin pathway to induce osteogenic differentiation of calvarial osteoblasts [18].

### 6.4. RANKL Signaling

RANKL, an important member of tumor necrosis factor (TNF) superfamily, is also known as osteoclast differentiation factor (ODF). The binding of extracellular signaling factor RANKL to RANK activates signaling cascades by recruiting adapter protein tumor necrosis factor receptor-associated factor 6 (TRAF6), which leads to multiple downstream events such as activation of MAPKs (ERK, p38, and JNK) and NF-*κ*B [9].

Numerous studies have found that quercetin inhibits the formation of osteoclast-like cells, bone resorption pit, and F-actin ring formation in murine macrophage RAW264.7 cells, human peripheral-blood mononuclear cells (PBMCs), or bone marrow macrophages treated with M-CSF and/or RANKL [95].

In RAW264.7 cells treated with RANKL, kaempferol was shown to abrogate RANKL-induced cells, the indicator for osteoclast differentiation. The downregulation of osteoclastogenic factors including RANKL, nuclear factor of activated T-cells cytoplasmic 1, and TRAF6 was also observed in kaempferol-treated cells [5].

The effects of rutin on the development and activity of osteoclasts *in vitro* were compared with the effects of 17*β*-estradiol. The anti-resorbing properties of rutin were mainly mediated by ER proteins through the inhibition of RANK protein or the activation of caspases [98]. On the other hand, the research *in silico* interaction of rutin with the RANK/RANKL system in diabetoporosis showed that the initial interaction of RANK with rutin will facilitate the bond of RANK to RANKL. This is in contrast to previous findings that rutin decreases the activity of RANKL [99].

Ginsenoside Rg3 is one of the most promising compounds of ginseng with numerous biological activities. Inhibition of osteoclast differentiation by ginsenoside Rg3 in RAW264.7 cells via RANKL, JNK, and p38 MAPK pathways was observed [22].

Crocin, an important compound from saffron crocus style and stigma, inhibited RANKL-induced phosphorylation of JNK in BMMs. The results suggested that the inhibitory effect of crocin on the differentiation of osteoclast precursors into mature osteoclasts may be mediated by the regulation of JNK phosphorylation [36].

### 6.5. NF-*κ*B Signaling

NF-*κ*B is a set of nuclear factors that bind to consensus DNA sequences called *κ*B sites and is essential for osteoclast formation and survival. Abnormal activation of NF-*κ*B signaling in osteoclasts is associated with excessive osteoclastic activity and observed in osteolytic conditions frequently. Modulators of NF-*κ*B signaling pathways have a great therapeutic potential in bone disease. NF-*κ*B signaling pathways are strictly regulated by cytokines such as RANKL, TNF-*α*, and IL-1, which differentially regulate classical and/or alternative NF-*κ*B pathways to maintain bone homeostasis in osteoclastic cells [100]. Curcumin, an important dietary nutraceutical compound derived from the Indian spice turmeric, has been shown to inhibit both NF-*κ*B activation and osteoclastogenesis induced by RANKL [100].

Several studies using genetically engineered mouse models suggested that the NF-*κ*B pathway plays a key role in RANKL-induced osteoclast development and function [9].

Both quercetin and kaempferol potentially protect the bone through their anti-inflammatory property on osteoblastic cells via inhibition of NF-*κ*B nuclear translocation [5, 9]. Similarly, rutin inhibits osteoclast formation by decreasing reactive oxygen species and TNF-*α* by inhibiting activation of NF-*κ*B [101].

Three compounds crocin [36], ginsenoside Rb1 [23], and ginsenoside Rb2 [24] showed their inhibition on RANKL-induced osteoclastogenesis by regulating NF-*κ*B signaling pathways.

### 6.6. Macrophage-Colony-Stimulating Factor

Osteoclasts are specialized bone-resorbing cells regulated by RANKL and M-CSF. When monocytes were stimulated with M-CSF, mature osteoclasts were formed, and quercetin decreased this osteoclastogenesis [102].

## 7. Food Functions

TCM nutrition is an ancient but burgeoning discipline, and its main goal is to use food as a means to achieve balance and harmony within the body [12]. From the point of food supplementation, the viewpoints of MFH plants conform to today's food requirements of returning to a natural and green healthy life and seem easy to accept [103]. Take some examples, the edible flowers such as chrysanthemum [104], the vegetable such as chicory [105], and wild edible plants such as purslane [106] are all distributed all over the world and have been consumed as food since ancient times.

According to the “Measures for the Administration of Food Production Licensing,” the State Administration for Market Supervision and Administration updated the “Catalogue of Food Production Licensing” in 2020 [107]. Food functions of the 20 MFH plants were discussed as the following catalogues: condiment (5 MFH plants), tea and related products (3 MFH plants), vegetable products (3 MFH plants), beverages (3 MFH plants), healthy food (3 MFH plants), fruit products (2 MFH plants), and processed food (1 MFH plant).

### 7.1. Condiments

Both clove and *A. officinarum* are normally served as seasoning in daily life. The effectiveness of pectin coatings enriched with clove essential oil was investigated to preserve bream fillets during refrigeration. The results showed that pectin coating along with clove oil was effective in inhibiting bacterial growth especially in Gram-negative bacteria [108].

*Z. schinifolium* is distributed in more than 20 provinces in China including Sichuan. Sichuan-*Z. schinifolium* are also a popular food additive and widely used in cooking with the history of more than 1000 years for both medicinal and economic values [109].

Saffron, stigmas of *C. sativus*, is one of the most precious spices used as a food colorant and flavoring agent and is widely consumed in culinary for its famous and unique color. Crocin is the typical carotenoid pigment of saffron that gives food a rich golden-yellow tinge [110], and crocin also plays an important role as a nutraceutical compound and exhibits antiosteoporosis effects according to our review.

*C. album*, normally called Chinese olive, is a tropical and semitropical fruit of the family Burseraceae, widely cultivated in Taiwan, Southeast China, and other regions of Asia. Some findings suggest that Chinese olive may ameliorate metabolic dysfunction in diabetic rats under high-fat diet challenge [111].

### 7.2. Tea and Related Products

Both the fruits and leaves of *M. alba* have nutritional and medicinal values and could be served as tea. Mulberry fruits with high concentrations of anthocyanins are favored by consumers as fruits because of their good taste, bright color, and high nutritional value [112]. Findings indicated that mulberry leaves and soybean are both good sources of melatonin and free tryptophan and can be applied to prepare high-melatonin pasteurized milk [113].

Mulberry leaves and mulberry fruits have different clinical efficiencies according to TCM, but they both showed antiosteoporosis effects on OVX models [47, 54]. The phenolic profile of mulberry leaves was characterized by the presence of a high number of flavonol derivatives, mainly glycosylated forms of quercetin and kaempferol [114]. LC-MS analysis also revealed that the contents of four flavonoid glycosides including kaempferol rhamnosylhexoside increased after digestion of mulberry fruits [115]. Another popular tea in China is made from chrysanthemum flower [116]. As a novel natural antioxidant, chrysanthemum could be used for the meat processing industry [117].

*Siraitia* fruits have been used as a natural sweetener for more than 300 years [80]. The research of *Siraitia* fruit extract supplementation on the chemical, microbial, and sensory properties of probiotic yogurt showed that it offers a promising option as a dietary supplement to produce novel dairy products that have high nutritional and bioactivity values [118].

### 7.3. Vegetable Productions

Purslane is a popular wild edible plant. Wild edible plants are gaining importance as they are potential sources of food due to their nutritional value, besides showing positive health effects and offering innovative applications in haute cuisine [106].

*H. cordata* is a popular vegetable in Asian countries [119] and is an important traditional Chinese medicine used in heat clearing and detoxifying, swelling and discharging pus, promoting diuresis, and relieving stranguria [120]. It was reported that fermented juice of *H. cordata* can improve diabetic symptoms by enhancing insulin sensitivity, reducing oxidative stress, and suppressing inflammation [121].

Chicory is a perennial herb and is cultivated worldwide. So far, chicory has been used mainly in animal feed but also in several cases in the food industry: as salad, for teas and tea blends, for coffee supplementation, and as a source for the inulin production. Nowadays, there is an increasing interest in chicory utilization for food production and supplementation. Some compounds present in chicory, such as kaempferol, inulin, and oligofructose may be considered as potential carriers of food functionality [105].

### 7.4. Beverage

Herbal teas or herbal drinks are traditional beverages that are prevalent in many cultures around the world. *P. vulgaris* L. is as a major plant in the Chinese traditional functional beverage Guangdong herbal tea for the treatment of fevers, diarrhea, and sore mouth [122].

*H. acerba* has long been used as traditional folk remedies for alcohol intoxication. The antihangover effect of *H. acerba* extract was examined in a randomized controlled crossover trial. The results suggest that a favorable effect of *H. acerba* beverage on alcohol hangovers might be associated with enhancing homeostatic regulation of inflammatory response [123].

Eighteen novel smoothie products containing sea buckthorn (25–50%) with other fruits and vegetables were analyzed. The results showed that sea buckthorn enriched the flavonols in smoothies and provided the most sensory attractive [124].

### 7.5. Healthy Food

The related research showed that wolfberry ameliorates osteoporosis in OVX mice by improving several important parameters including BMC, BMD, and bone-turnover markers such as osteocalcin and calcium levels in serum [37]. Wolfberry has been used as a tonic medicine and a long-term healthy food, which can be served as nutritional soup [125]. Several clinical studies in healthy subjects show that consumption of wolfberry juice improves general wellbeing and immune functions [74].

As another nutrition is this study, the safety and effectiveness of ginseng in different regions including American ginseng, Korean ginseng, and Asian ginseng were supported by some research [126] and already accepted in the East and West. Ginseng can be served as mixed wine, tea blends, alternative tea, soup, etc [107].

*P. sibiricum* is widely distributed in most regions in the south of the Yangtze River in China. It has served as a Taoist health potion such as soup and food ingredient since ancient times and functions to nourish the liver and kidney and prolong life [127].

## 8. Conclusive Remarks

Based on the literature research, 20 MFH plants are discussed in this review. Their common features are they all contain quercetin, rutin, and kaempferol and they all showed antiosteoporosis activities, *in vivo* and *in vitro.* The TCM characteristics including natures, flavors, attributive to meridian tropism, and efficiencies of the 20 MFH plants are compared. We have tried to explain their traditional efficiencies with pharmacological actions. Based on antiosteoporosis pathway research of quercetin, rutin, and kaempferol, more mechanisms of the 20 MFH plants should be evidenced for further application. At the same time, how to evaluate the actions of these compounds in the MFH plants is another problem that should be solved. Anyway, as food and food supplementation, these MFH plants with multiple nutraceutical compounds can be served as multiple food forms in our daily life. This is the focus of our concern.

## Figures and Tables

**Table 1:** The list of 20 medicine food homology plants and their TCM characteristics.

Classification in TCM^&^	Botanical name^*∗*^	English name^#^(Chinese name^*∗*^)	Nature^*∗*^	Flavor^*∗*^	Attributive to meridian tropism^*∗*^	Efficiency in TCM^*∗*^
Heat-clearing CMHs	*Portulaca oleracea*	Purslane (Machixian)	Cold	Sour	Intestine, liver, and spleen	Clears away heat and relieves toxins, cools the blood, and stops bleeding.
	*Houttuynia cordata*	Heartleaf Houttuynia herb (Yuxingcao)	Slight cold	Pungent	Lung	Clears away heat and relieves toxins, treats carbuncle and promotes pus drainage, and promotes diuresis to treat stranguria.
	*Prunella vulgaris*	Common selfheal spike (Xiakucao)	Cold	Bitter and pungent	Liver and gallbladder	Clears away liver-fire and disperses stagnation.
	*Canarium album*	Immature Tomentosa Terminalia (Qingguo)	Cool	Sweet and sour	Stomach and lung	Clears away heat and relieves toxins, eases the throat, and resolves phlegm.
	*Hovenia acerba*	Raisin tree seed (Zhijuzi)	Neutral	Sweet and bitter	Stomach	Clears away heat and promotes diuresis, and relieves alcohol toxins.
	*Cichorium intybus*	Chicory (Juju)	Cool	Bitter and salty	Spleen, liver, and bladder	Clears away liver-fire, relieves constipation, and promotes diuresis.

Tonics	*Panax ginseng*	Ginseng (Renshen)	Neutral	Sweet and slightly bitter	Spleen, lung, and heart	Invigorates renal *qi*, strengthens *qi* of the spleen and lung, promotes production of the body fluids to quench thirst, and calms the mind to promote intelligence.
	*Morus alba*	Mulberry fruit (Sangshen)	Cold	Sweet	Heart, liver and kidney	Nourishes *yin* and enriches the blood, nourishes the liver, and invigorates the kidney.
	*Polygonatum sibiricum*	Siberian Solomon's seal rhizome (Huangjing)	Neutral	Sweet	Spleen, lung, and kidney	Moistens the lung and nourishes the kidney, invigorates the kidney, and benefits *qi*.
	*Lycium barbarum*	Wolfberry fruit (Gouqizi)	Neutral	Sweet	Liver and kidney	Tonifies the kidney and benefits essence, and nourishes the liver and improves eyesight.
	*Rubus chingii*	Palmleaf raspberry fruit (Fupenzi)	Warm	Sweet and sour	Liver and kidney	Benefits the kidney to preserve the essence and reduces the frequency of urination, and nourishes the liver to treat eye diseases.

The interior warming CMHs	*Eugenia caryophyllata*	Clove (Dingxiang)	Warm	Pungent	Spleen, stomach, and kidney	Warms the middle energizer to low the adverse rising *qi*, and promotes circulation of *qi* and alleviates pain.
	*Zanthoxylum schinifolium* /*Zanthoxylum bungeanum*	Bunge pricklyath pericarp (Huajiao)	Hot	Pungent	Spleen, stomach, and kidney	Warms the middle energizer to alleviate pain.
	*Alpinia officinarum*	Lesser galangal rhizome (Gaoliangjiang)	Hot	Pungent	Spleen and stomach	Expels cold and relieves pain, and warms the spleen and stomach to stop vomiting.

Diaphoretics with pungent-cool property	*Morus alba*	Mulberry leaf (Sangye)	Cold	Bitter and sweet	Lung and liver	Expels wind and heat, clears away lung heat and moisturizes dryness, and clears away liver-fire to treat eye diseases.
	*Chrysanthemum morifolium*	Chrysanthemum flower (Juhua)	Cold	Bitter and pungent	Lung and liver	Expels wind and clears away heat, clears liver-fire to treat eye diseases, and eliminates toxic substances.

Phlegm resolving, antitussive and antiasthmatic CMHs	*Siraitia acerba*	Siraitia fruit (Luohanguo)	Cool	Sweet	Lung and spleen	Resolves phlegm and arrests cough, clears away lung heat and eases throat, and moistens the intestine to relieve constipation.
	*Hippophae rhamnoides*	Sea buckthorn fruit (Shaji)	Warm	Sour and astringent	Lung, spleen, stomach, and liver	Resolves phlegm and arrests cough, and nourishes the spleen and stomach.

CMHs for invigorating the blood and removing blood stasis	*Crocus sativus*	Saffron crocus style and stigma (Xihonghua)	Warm	Pungent	Heart and liver	Promotes blood circulation to remove blood stasis, promotes menstruation, and alleviates pain.

Dampness-removing CMHs	*Coix lacryma-jobi*	Adlay (Yiyiren)	Cold	Sweet	Spleen, stomach, and lung	Promotes diuresis to resolve dampness and invigorates the spleen, treats *Bi*-syndrome, and clears away heat to drain the pus.

*Notes*. ^*∗*^Botanical name, Chinese name, natures, flavors, attributive to meridian tropism, and efficiency in TCM are according to Chinese Pharmacopoeia (Chinese Pharmacopoeia Commission, China Medical Science Press, Beijing, China, 2020). ^#^English name is according to the Chinese Herbal Medicine Name Dictionary (Z. W. Xie, Beijing Science and Technology Press, Beijing, China, 2004). ^&^Classification in TCM is according to the Science of Chinese Materia Medica (D. C. Tang and J. Y. Xun, Publishing House of Shanghai University of TCM, Shanghai, China, 2003). Abbreviations: TCM, traditional Chinese medicine; CMHs, Chinese medicine herbs.

**Table 2:** *In vitro* research of the 20 medicine food homology plants on osteoblastogenesis and osteoclastogenesis process.

Botanical name^*∗*^	On osteoblastogenesis process	On osteoclastogenesis process	References
*Portulaca oleracea*	Purslane on human osteoblasts.	(1) The extract on RANKL-induced primary mice BMMs.	[13–15]
		(2) The extract on primary mice osteoclast cells.	

*Prunella vulgaris*	The extract on glucocorticoids-induced BMMSCs by activating the Smad pathway.		[16]
*Canarium album*		The extract on RANKL-induced RAW264.7 cells.	[17]
*Hovenia acerba*	The extract on calvarial osteoblasts from the calvaria of ICR mice at postnatal day, via Wnt/*β*-catenin pathway.		[18]

*Panax ginseng*	(1) Ginsenoside Rb1 on aluminum chloride-induced rat osteoblasts.	(1) Ginsenoside Rb1 on RANKL-induced RAW264.7 cells, via NF-*κ*B and MAPKs pathways	[19–24]
	(2) Ginsenoside Rb2 on hydrogen peroxide-induced osteoblastic MC3T3-E1 cells, via reduction of oxidative damage.	(2) Ginsenoside Rb2 on RANKL-induced RAW264.7 cells, via NF-*κ*B and STAT3	
	(3) Ginsenoside Rg3 on osteoblastic MC3T3-E1 cells.	(3) Ginsenoside Rg3 on RANKL-induced RAW264.7 cells, via RANKL, JNK, and p38 MAPK pathways.	

*Polygonatum sibiricum*	The polysaccharide on primary mice BMMSCs, via ERK/GSK‐3*β*/*β*‐catenin and Wnt/*β*-catenin pathways.	The polysaccharide on RANKL-induced primary mice BMMs.	[25–27]
*Lycium barbarum*	The polysaccharides on the osteoblast MC3T3‐E1 cell line.		[28]
*Rubus chingii*	Seven compounds on primary rat osteoblasts.	Seven compounds on primary rat osteoclasts.	[29]
*Zanthoxylum schinifolium* /*Zanthoxylum bungeanum*		The extract on RANKL-induced RAW264.7 cells.	[17]
*Alpinia officinarum*	The extract on primary rat osteoblasts isolated from newborn rat calvariae.	The extract on RANKL-induced primary mice BMMs.	[30, 31]
*Chrysanthemum morifolium*	Linarin on the osteoblast MC3T3‐E1 cell line, via BMP-2/Runx2 pathway.		[32]
*Siraitia acerba*	Mogroside V on primary rat osteoblasts isolated from newborn rat calvariae.		[33]

*Crocus sativus*	(1) Crocin on the osteoblast MC3T3‐E1 cell line.	Crocin on RANKL‑induced primary mice BMMs, via JNK and NF‑*κ*B pathways.	[34–36]
	(2) Crocin and crocetin on primary rat BMMSCs.		

*Coix lacryma-jobi*	(1) The extract on the primary rat osteoblasts isolated from the calvaria of fetal rats (18 days old) via the ERK‑regulated pathway.		[37, 38]
	(2) The water extract of adlay seeds in cultured neonatal rat calvariae.		

*Notes*. ^*∗*^Botanical name are according to Chinese Pharmacopoeia (Chinese Pharmacopoeia Commission, China Medical Science Press, Beijing, China, 2020). Abbreviations: RANKL, receptor activator for nuclear factor *κ*B ligand. BMMs, bone marrow-derived macrophages; BMMSCs, bone marrow mesenchymal stem cells; ICR, Institute of Cancer Research; NF-*κ*B, nuclear factor kappa B; MAPK, mitogen-activated protein kinases; p38 MAPK, p38 mitogen-activated protein kinase; STAT3, signal transducer and activator of transcription protein 3; JNK, c-Jun N-terminal kinase; ERK, extracellular signal regulated kinase; GSK‐3*β*, glycogen synthase kinase-3*β*; Wnt, wingless; BMP-2, bone morphogenetic protein 2; Runx2, runt-related transcription factor 2.

**Table 3:** *In vivo* animal research of the 15 medicine food homology plants.

Botanical name^*∗*^	Model	Dose	Route	Intervention time	Main improved results	Reference
*Portulaca oleracea*	The extract in LPS-induced osteolysis male mice.	250 mg/kg	Administered orally	Every 2 days for 8 days	Bone loss, bone erosion, and the number of TRAP-positive osteoclasts↓	[13]
					BV/TV, Tb.Sp, and Tb.N↑	

*Houttuynia cordata*	The essential oil in OVX mice.	10 and 20 mg/kg	I.g.	12 weeks	ALP, TRAP, TNF-*α*, IL-1*β*, and MDA↓	[39]
					SOD, parameters of bone morphometry , and biomechanical properties↑	

*Prunella vulgaris*	The flavonoids in OVX rats.	10%	I.g.	12 weeks	ALP, the number of osteoclasts, and bone resorption perimeter percentage↓	[40]
					OPG, BMD, and the relative volume and thickness of trabecular bone↑	

*Hovenia acerba*	(1) The extract in normal 8-week-old male mice.	200 mg/kg	I.p.	5 sequential days each week for 4 weeks	Trabecular bone, BV/TV and Tb.N, femoral bone mass, and thickness and area of femoral cortical bone↑	[18]
					Trabecular or cortical femoral bone loss↓	
	(2) Methyl vanillate in OVX mice.		Administrated orally	5 sequential days each week for 4 weeks	BV/TV, Tb.N, trabecular bone volume↑	

*Cichorium intybus*	(1) Chicory inulins and a mixture of inulins-isoflavones in OVX rats.	385 mg/day	In water	2 months	BMD↑	[41]
	(2) Chicory inulin in growing male rats.	5 and 10 g/100 g diet	In diet	22 weeks	WBBMC and WBBMD↑	[42]
	(3) A purified native inulin, a reformulated inulin, and a dehydrated chicory in young male rats.	7.5% inulin in the diet	In diet	3 months	Mg absorption, BMD, and breaking load↑	[43]
	(4) The chicory extracts in GIOP rats.	100 mg/kg	Administrated orally	3 times per week for 8 weeks	Ca, P, BMD, BMC↑	[44]
					PTH, ALP↓	

*Panax ginseng*	(1) Ginseng on osteoporosis in OVX rats in which inflammation was induced.	100 and 200 mg/kg	Administrated orally	20 days	BMD↑	[45]
					OC, TNF-*α*, IL-1*β*, IL-6↓	
	(2) Ginsenoside Rg3 in OVX rats	20 mg/kg	I.p.	Every 2 days for 5 weeks	Thickness, number, and density of trabeculae, osteogenesis↑	[19]
	(3) Ginseng extracts in 112-week-old male rats.	300 mg/kg/day	Administrated orally	8 weeks	Total BMD in the tibia，osteoblast↑	[46]

*Morus alba*	The extract in OVX rats.	0.5% or 1%	Tube feeding	8 weeks	ALP, fragile structure was reduced↓	[47]
					Trabecular thickness↑	

*Polygonatum sibiricum*	(1) The polysaccharide in OVX rats.	100, 200, and 400 mg/kg	I.g.	Every 2 days for 35 days	BMD, BGP↑	[48]
					ALP, TRAP, and TNF-*α*↓	
	(2) The polysaccharide on osteoporotic fracture, which is established by OVX rats.	100, 500, and 1000 mg/kg	I.g.	8 weeks	TRAP and PINP↓	[49]
					ALP, OPG, the maximum tibial load, elastic load, BMD, BGP, GPR48, and BMP-2 protein↑	

*Lycium barbarum*	The extract in OVX mice.	1 and 100 mg/kg	Administrated orally	6 weeks.	BMC, BMD, CON, calcium↑	[50]
					Hypertrophy of the epiphyseal plate ↓	

*Eugenia caryophyllata*	The extract in OVX rats.		Administrated orally	4 weeks	AP, TRAP, urinary phosphate, and creatinine↓	[51]
					Ca, bone density, bone mineral content, bone tensile strength↑	

*Alpinia officinarum*	The extract in OVX rats.	300 mg/kg	I.g.	12 weeks	OP↓	[30]
					Bone strength↑	

*Crocus sativus*	(1) Crocin in OVX rats.	5, 10, and 20 mg/kg	Administrated orally	16 weeks	BMD of L4 vertebrae and femurs, skeletal remodeling, bone-turnover markers↑	[52]
					Oxidative stress status in bone tissue↓	
	(2) Crocin in metabolic syndrome-induced osteoporosis rats.	5 and 10 mg/kg	I.g.	5 sequential days each week for 12 weeks	OCN, longitudinal, and perpendicular forces of femurs↑	[53]
					TRAP, CTX1, IL-6, TNF-*α*, oxidative stress in femur distal epiphysis tissues↓	

*Morus alba*	The combined extract of mulberry leaf and *Polygonum odoratum* in OVX rats.	5, 150, and 300 mg/kg	Administrated orally	3 months	Oxidative stress and osteoclast density↓	[54]
					Osteoblast density and cortical thickness, serum Ca, ALP, and OCN↑	

*Chrysanthemum morifolium*	Linarin in OVX mice	50 and 150 mg/kg	I.g.	8 weeks	BMD, BV/TV, BS/TV, and Tb.N↑	[32]
					ALP and OCN↓	

*Coix lacryma-jobi*	(1) The adlay diet and adlay extract in OVX mice.	10% and 30% in diet	In diet	4 weeks	ALP, Ca, and BMD↑	[37]
	(2) The extract in OVX rats.	300 *μ*g/mL	Administrated orally	4 weeks	ALP, Ca↑	[38]
					TRAP↓	

*Notes*. ^*∗*^Botanical name are according to Chinese Pharmacopoeia (Chinese Pharmacopoeia Commission, China Medical Science Press, Beijing, China, 2020). Abbreviations: ALP, alkaline phosphatase; BMD, bone mineral density; BGP, bone Gla protein; BMP-2, bone morphogenetic protein-2; BV/TV, bone volume/total volume; CTXI; collagen cross-linking carboxy-terminal telopeptide, type I; GIOP, glucocorticoid-induced osteoporosis; GPR48, G protein-coupled receptor 48; IL-1*β*, interleukin-1*β*; LPS, lipopolysaccharide; MDA, malondialdehyde; OCN, osteocalcin; OP, osteopontin; OPG, osteoprotegerin; OVX, ovariectomy; PINP, procollagen type I N-terminal propeptide; PTH, parathyroid hormone; SOD, superoxide dismutase; Tb.N, trabecular number; Tb.Sp, trabecular separation; TNF-*α*, tumor necrosis factor-*α*; TRAP, tartrate-resistant acid phosphatase; WBBMC, whole-body bone mineral content; WBBMD, whole-body bone mineral density.

## Data Availability

All the authors declare that the readers can access the conclusions from the five figures and three tables. All the figures and tables are summarized based on the references.

## Abbreviations

APC: Adenomatosis polyposis coli
AXIN: Axis inhibition protein 2
BMC (g/cm): Bone mineral content
BMD (g/cm^2^): Bone mineral density
BMMs: Bone marrow-derived macrophages
BMMSCs: Bone marrow mesenchymal stem cells
BMPs: Bone morphogenetic proteins
CK1: Casein kinase 1 alpha
ER: Estrogen receptor
ERK: Extracellular signal regulated kinase
FGF: Fibroblast growth factor
FGFRs: FGF tyrosine kinase receptors
GIOP: Glucocorticoid-induced osteoporosis
GSK3*β*: Glycogen synthase kinase-3 beta
JNK: C-Jun n-terminal kinase
LDL: Low-density lipoprotein
LPS: Lipopolysaccharide
MAPKs: Mitogen-activated protein kinases
M-CSF: Macrophage-colony-stimulating factor
MFH: Medicine food homology
NF-*κ*B: Nuclear factor kappa B
ODF: Osteoclast differentiation factor
OVX: Ovariectomy
PBMCs: Human peripheral-blood mononuclear cells
PTH: Parathyroid hormone
RANKL: Receptor activator for nuclear factor *κ*B ligand
RUNX2: Runt-related transcription factor 2
SMAD: Suppressor of mothers against decapentaplegic
STAT3: Signal transducer and activator of transcription protein 3
TCM: Traditional Chinese medicine
TGF-*β*: Transforming growth factor-*β*
WNT: Wingless
TNF: Tumor necrosis factor
TRAF6: Tumor necrosis factor receptor-associated factor 6.

## Additional Points

Chemical compounds mentioned in this article: Crocetin (PubChem CID: 5281232); Crocin (PubChem CID: 5281233); Curcumin (PubChem CID: 969516); Ginsenoside Rb1 (PubChem CID: 9898279); Ginsenoside Rb2 (PubChem CID: 6917976); Ginsenoside Rg3 (PubChem CID: 9918693); Inulin (PubChem CID: 24763); Kaempferol (PubChem CID: 5280863); Linarin (PubChem CID: 5317025); Quercetin (PubChem CID: 5280343); Rutin (PubChem CID: 5280805); Resveratrol (PubChem CID: 445154); Genistein (PubChem CID: 5280961).
